# Pathophysiology of and therapeutic options for a *GABRA1* variant linked to epileptic encephalopathy

**DOI:** 10.1186/s13041-019-0513-9

**Published:** 2019-11-10

**Authors:** Yun-Fei Bai, Michelle Chiu, Elizabeth S. Chan, Peter Axerio-Cilies, Jie Lu, Linda Huh, Mary B. Connolly, Ilaria Guella, Matthew J. Farrer, Zhi-Qing David Xu, Lidong Liu, Michelle Demos, Yu Tian Wang

**Affiliations:** 1 Djavad Mowafaghian Centre for Brain Health and Department of Medicine, University of British Columbia, Vancouver, Canada; 2 Department of Neurobiology, Beijing Key Laboratory of Neural Regeneration and Repair, Beijing Laboratory of Brain Disorders (Ministry of Science and Technology), Beijing Institute for Brain Disorders, Capital Medical University, Beijing, China; 3 Division of Neurology, Department of Paediatrics, BC Children’s Hospital, University of British Columbia, Vancouver, Canada; 4 Centre for Applied Neurogenetics, University of British Columbia, Vancouver, Canada; 5 McKnight Brain Institute, University of Florida, Gainesville, USA

**Keywords:** GABA a receptor, Epileptic encephalopathy, Mutation, Therapeutic options

## Abstract

We report the identification of a de novo *GABRA1* (R214C) variant in a child with epileptic encephalopathy (EE), describe its functional characterization and pathophysiology, and evaluate its potential therapeutic options. The *GABRA1* (R214C) variant was identified using whole exome sequencing, and the pathogenic effect of this mutation was investigated by comparing wild-type (WT) α1 and R214C α1 GABA_A_ receptor-expressing HEK cells. GABA-evoked currents in these cells were recorded using whole-cell, outside-out macro-patch and cell-attached single-channel patch-clamp recordings. Changes to surface and total protein expression levels of WT α1 and R214C α1 were quantified using surface biotinylation assay and western blotting, respectively. Finally, potential therapeutic options were explored by determining the effects of modulators, including diazepam, insulin, and verapamil, on channel gating and receptor trafficking of WT and R214C GABA_A_ receptors. We found that the *GABRA1* (R214C) variant decreased whole-cell GABA-evoked currents by reducing single channel open time and both surface and total GABA_A_ receptor expression levels. The GABA-evoked currents in R214C GABA_A_ receptors could only be partially restored with benzodiazepine (diazepam) and insulin. However, verapamil treatment for 24 h fully restored the function of R214C mutant receptors, primarily by increasing channel open time. We conclude that the *GABRA1* (R214C) variant reduces channel activity and surface expression of mutant receptors, thereby contributing to the pathogenesis of genetic EE. The functional restoration by verapamil suggests that it is a potentially new therapeutic option for patients with the R214C variant and highlights the value of precision medicine in the treatment of genetic EEs.

## Introduction

Epileptic encephalopathy (EE) is a severe neurological condition in which a patient’s epileptic activity results in additional cognitive or behavioral impairments beyond those expected from the underlying etiology alone [1]. Growing evidence demonstrates that pathogenic genetic variants are a common risk factor for EE, including variants in the γ-aminobutyric acid type A (GABA)_A_ receptor, the principle receptor that mediates the inhibitory synaptic transmission in the mammalian brain [2–21]. GABA_A_ receptors (GABA_A_Rs) are pentameric chloride channels assembled from several families of subunits, including α_1–6_, β_1–3_, γ_1–3_, δ, ε, θ, π and ρ [22–25]. The most common native GABA_A_R at the inhibitory synapse is composed of two α1, two β2 and one γ2 subunits [22–27]. These subunits contain a large extracellular N-terminal domain, four transmembrane (TM) (TM1–4) segments, a small and a large intracellular loop domain, and a short extracellular C-terminal domain [22–25, 28] Proper assembly of these subunits in the endoplasmic reticulum (ER) is required to form functional GABA_A_Rs and to target GABA_A_Rs to specific subcellular domains in neurons [29, 30].

The α1 subunit is encoded by the *GABRA1* gene and is abundantly expressed in most brain regions [18, 29]. *GABRA1* variants were first identified in patients with idiopathic generalized epilepsy, specifically juvenile myoclonic epilepsy, childhood absence epilepsy, and generalized epilepsy with febrile seizures plus [5, 15–17]. More recently, *GABRA1* variants have been associated with severe phenotypes such as Dravet Syndrome and early-onset EEs, as well as with variable degrees of developmental delay, behavioral problems and autistic features [4, 13, 14]. The most common seizure types are myoclonic and generalized tonic-clonic seizures. EEG recordings show generalized sharp waves in almost all patients and photoparoxysmal response in approximately 50% of these patient s[13].

Functional studies have revealed that these mutations may contribute to pathogenesis of disease through haploinsufficiency of GABA_A_R-mediated neuronal inhibition as a result of reduced numbers of receptors on the plasma membrane surface (due to decreased protein stability and plasma membrane trafficking) or receptor function (due to impaired channel gating properties) or a combination of the two. The diminished GABA_A_R-mediated inhibition in turn leads to increased neuronal excitability, thereby contributing to epileptopathogenesis [5, 17, 21].

We identified a de novo *GABRA1* (R214C) variant in a patient with EE. Using a heterologous HEK293 cell system, we characterized the functional impact of the mutation and its underlying pathogenic mechanisms. We found that the R214C α1 variant significantly decreased GABA-evoked whole-cell current amplitudes due to a combination of decreased receptor expression and compromised channel activity.

We explored potential therapeutic options for R214C GABA_A_Rs. We demonstrated that increasing channel activity with diazepam [31] and increasing cell surface receptor expression with insulin, which was previously reported to promote a rapid translocation of GABA_A_Rs from intracellular compartments to the plasma membrane surface, [32] both enhanced the function of R214C GABA_A_Rs. However, even a combination of insulin and diazepam only achieved a partial rescue of currents gated through the mutant receptor. In contrast, we found that verapamil, a L-type calcium channel blocker that has recently been reported to improve receptor folding and surface expression of a recombinant GABA_A_R containing a D219N variant, [33] could fully rescue currents gated through the mutant receptor to the same level as WT GABA_A_Rs. Our study highlights the importance of functional and pharmacological characterization of genetic variants, and the potential of precision medicine in the management of early-onset EE.

## Materials and methods

### Genetic analysis

This work was approved by site-specific Institutional Review Boards and informed consent was obtained before study inclusion (H14–01531). The patient was identified through the Epilepsy Genomics Study (EPGEN) at BC Children’s Hospital, a clinical study assessing the yield of targeted whole-exome sequencing (WES) in children with early-onset epilepsy of unknown cause.

Peripheral blood samples were collected from the proband and her parents. Genomic DNA was extracted from peripheral blood lymphocytes following standard protocols. Exonic regions were captured using the Ion AmpliSeq Exome Kit (57.7 Mb) and WES was performed on the Ion Proton System according to manufacturers’ recommendations (Life Technologies, Carlsbad, CA). Analysis was restricted to 620 genes previously implicated in epilepsy. Candidate variants were validated by Sanger sequencing as previously described [34]

### Clinical phenotype

The patient’s clinical evolution, EEG and neuroimaging were described, and seizures were classified according to the International League Against Epilepsy Organization [35].

### Homology modeling of the GABA_A_R

The homology model of the most abundant subtype of the α1β2γ2 GABA_A_R was constructed by using methods described elsewhere [36]. This protocol uses the x-ray structure of GluCl co-crystallized with glutamate (PDB code 3RIF) as the primary template for homology modeling [37]. The model was constructed using MODELLER 9v7 [38]. A second homology model of the α1β2γ2 subtype was also built using the recent crystal structure of a human gamma-aminobutyric acid receptor, the GABA_A_R-β3 homopentamer (PDB code 4COF) as the template [39]. Structure validation was performed using VERIFY-3D [40] on the SWISS-PDB server. Molecular graphics and analyses were performed with UCSF Chimera, which was developed by the Resource for Biocomputing, Visualization, and Informatics at the University of California, San Francisco [41].

### Complementary DNA constructs

The cDNAs encoding rat GABA_A_R α1, β2 and γ2 subunits and EGFP were cloned into pcDNA3.0 expression vectors (Invitrogen). The novel variant mutant α1 (c.640C > T) subunit constructs were generated by gene specific primers with fusion polymerase chain reaction (PCR) and confirmed by DNA sequencing.

### Cell culture and transfection

HEK293 cells were maintained in Dulbecco’s Modified Eagle Medium (DMEM; Sigma) supplemented with 10% fetal bovine serum (FBS; Invitrogen) at 37 °C in a 5% CO_2_ incubator. For electrophysiology experiments, cells were grown to ~ 80% confluence in six-well plates and transiently transfected with rat cDNAs encoding α1:β2:γ2 (1 μg:1 μg:0.5 μg) or α1(R214C):β2:γ2 (1 μg:1 μg:0.5 μg) GABA_A_R subunits using lipofectamine 2000 (Invitrogen) according to the manufacturer’s instruction. EGFP cDNA (0.25 μg) was also co-transfected with GABA_A_R subunits to serve as an indicator for successfully transfected cells during electrophysiological recordings. HEK293 cells were re-plated onto poly-L-lysine-coated 22-mm glass coverslips in 24-well dishes after transfection for 24 h and cultured for an additional 24–48 h before recording. For western blot assay to study total and surface protein expression, cells were grown to ~ 70% confluence in six-well plates and transiently transfected with rat cDNAs encoding α1:β2:γ2 (1 μg:1 μg:0.5 μg) or α1(R214C):β2:γ2 (1 μg:1 μg:0.5 μg) GABA_A_R subunits.

### Western blot and surface biotinylation

Transfected HEK293 cells were washed with ice-cold PBS three times, and lysed with 10% SDS-containing cocktail protease inhibitor (Bimake, Huston, USA) mixture at 4 °C for 30 min. The supernatant was collected by centrifugation (13,000 g, 20 min, 4 °C) and protein concentration was measured by MicroBCA assay (Biorad, California, USA). The protein samples were cleaved by six times sample buffer containing 9% beta-mercaptoethanol and boiled at 65 °C for 5 min before loading onto 10% SDS-PAGE gels. Proteins were transferred to PVDF membranes (EMD Millipore, Burlington, USA) and anti-α1 subunit antibody (1:1000) (EMD Millipore) was used to detect WT and variant GABA_A_R α1 subunits. β-actin (antibody 1:3000, Sigma) served as a loading control for total proteins. Band intensity was quantified using ImageJ software (NIH).

In biotinylation assays, cells were harvested 48 h post-transfection and washed with ice-cold PBS three times (5 min each) before incubating with the membrane-impermeable reagent Sulfo-HNS-LC-Biotin (1 mg/ml, Thermo Scientific) at 4 °C for 30 min to label surface membrane proteins. To quench the reaction, cells were washed with 100 mM glycine dissolved in ice-cold PBS three times (5 min each) at 4 °C. Cells were solubilized for 30 min at 4 °C in lysis RIPA buffer (150 mM NaCl, 1% Triton X-100, 0.5% Sodium deoxycholate, 0.1% SDS and 50 mM Tris-HCl, pH = 8) supplemented with cocktail protease inhibitor mixture (Bimake, Huston, USA). The supernatant containing the biotinylated surface proteins were collected by centrifugation (13,000 g, 20 min at 4 °C). The protein concentrations were measured using BCA assay (Biorad). The biotin-labeled plasma membrane proteins were incubated with High Binding Capacity NeutrAvidin beads (Thermo Scientific) overnight and were pulled down with the beads after centrifugation. The samples were lysed by 10% SDS containing cocktail protease inhibitor mixture (Bimake) and cleaved by six times sampling buffer (Invitrogen) containing 9% beta-mercaptoethanol. The protein samples were boiled at 65 °C, 5 min and loaded onto 10% SDS-PAGE gels. The Na^+^/K^+^ ATPase (antibody 1:1000, Abcam) served as a loading control for biotinylated membrane proteins.

### Electrophysiology

Whole-cell, outside-out and cell-attached single channel recordings of WT and R214C GABA_A_R currents were performed at room temperature on transfected HEK293 cells as previously described [32, 42]. For whole-cell and outside-out recordings, the extracellular solution (ECS) contained (in mM): 130 NaCl, 5 KCl, 2 CaCl_2_, 2 MgCl_2_, 10 HEPES, 10 glucose and 10 sucrose (pH = 7.4, 300–310 mOsm). The patch pipettes (3–5 MΩ) were made from thin-walled borosilicate glass (World Precision Instruments, USA) with a micropipette puller (Sutter Instruments, model P-97, Novato, CA). The internal solution contained (in mM): 140 CsCl, 0.1 CaCl_2_, 2 MgCl_2_, 10 HEPES, 10 BAPTA and 4 ATP (K) (pH = 7.2, 290–300 mOsm). The Cl^−^ reversal potential was near 0 mV under recording condition with the above intra/extra-cellular solutions, and cells were voltage clamped at -60 mV. Current amplitudes in whole-cell recording were obtained by applying GABA (0.1–1000 μM) through a computer-controlled fast step perfusion system (Warner Instruments) for 1 s. GABA_A_R current kinetics including activation, deactivation and desensitization time constants (τ) were obtained by application of 10 mM GABA for 400 ms. For current-voltage (I/V) relation experiments, GABA (1 mM, 1 s) evoked currents were recorded by holding the cell membrane potential (in mV) at: − 80, − 60, − 40, − 20, 0, + 20, + 40 and + 60.

Cell-attached single channel recordings were obtained in an external solution containing (in mM): 140 NaCl, 5 KCl, 1 MgCl_2_, 2 CaCl_2_, 10 glucose and 10 HEPES (pH = 7.4, 300–310 mOsm). The electrodes were polished to a resistance of 10–20 MΩ and filled with solution containing (in mM): 120 NaCl, 5 KCl, 10 MgCl_2_, 0.1 CaCl_2_, 10 glucose, 10 HEPES and 1 GABA (pH = 7.4, 300–310 mOsm), and holding potential was held at + 100 mV.

Whole-cell, outside-out and single channel currents were low-pass filtered at 2 kHz using an Axopatch 200B amplifier (Axon Instruments), digitized at 10 kHz (whole cell and outside-out recordings) or 20 kHz (cell-attached single channel recordings) using Digidata 1322A, and recorded using Clampex 10.3 (Axon Instruments, Sunnyvale, CA). Data were analyzed offline using Clampfit 10.3 (Axon Instruments) as previously described [32, 42].

Single channel open and closed events were analyzed using the 50% threshold detection method and visually inspected before accepting the events. Single channel open probability was determined by the total amount of channel open time within the analyzed time. Total closed time was determined as the difference between total open time and total analyzed time.

### Chemicals

Diazepam (Sandoz, Quebec, Canada) was diluted in ECS from stock solution to 1 μM in electrophysiology experiments. Insulin (Sigma Aldrich, USA) was weighed and dissolved directly in ECS to form a 0.5 μM solution in electrophysiology experiments. Verapamil (Tocris, Bristol, UK) was diluted in water to a make 4 mM stock. In acute electrophysiological recordings, verapamil was diluted (1:1000) in ECS and perfused onto cells. In 24 h treatment experiments, verapamil was diluted (1:1000) in DMEM.

### Data analysis

Data were presented as mean ± SEM (n = number of cells). The two-way ANOVA (followed by post hoc Student’s t test), paired or unpaired (two-tailed) Student’s *t* test were used for statistical analysis and *p* < 0.05 was considered statistically significant. Dose-response curves were fitted by *Hill* equation and EC_50_ was calculated by GraphPad prism 6. Whole-cell peak currents, channel gating and kinetic properties and single-channel currents were analyzed by Clampfit 10.3.

### Data availability

Data supporting our findings are found within the article and in the Additional file 1: Figure S1.

## Results

### Clinical phenotype and genotype of a patient with EE

**Fig. 1:**
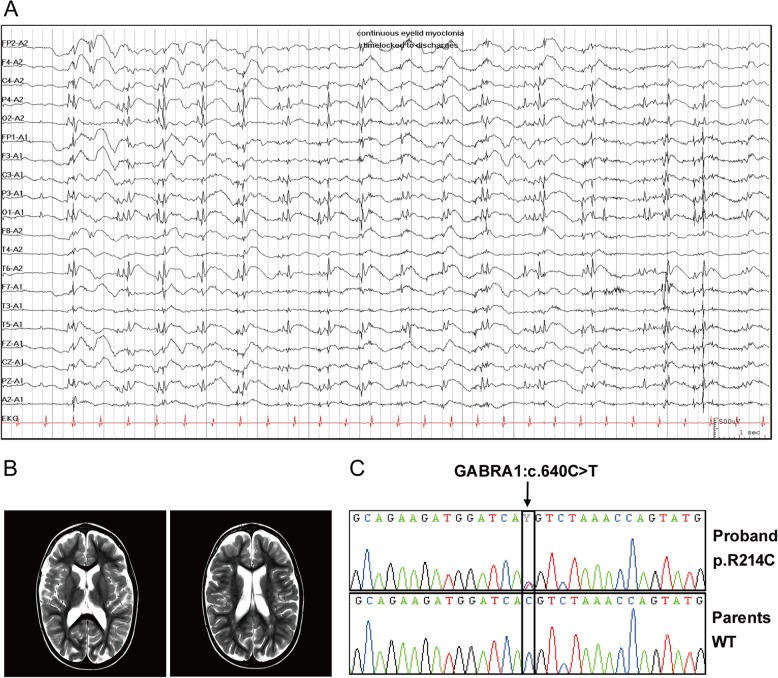
EEG and MRI abnormality and genotype of a patient with epileptic encephalopathy (EE). **a** Ictal EEG shown in a referential montage demonstrates rhythmic generalized and bi-posterior quadrant spike and wave and polyspike and wave discharges which were time-locked with eyelid myoclonia. **b** T2-weighted MRI brain at age 4 demonstrates mild periventricular leukomalacia and non-progressive ventriculomegaly. **c** Sanger confirmation of variant, including confirmation of absence from both biological parents, was performed. These electropherograms illustrate the proband’s GABRA1 c.640C > T; p.Arg214Cys pathogenic variant compared with that of her parents (wild type)

The patient is an 11-year-old girl with EE, treatment-resistant epilepsy, intellectual disability, and autism spectrum disorder. Seizure onset was at 11 months. Initially, she had focal motor seizures with impaired awareness which progressed to bilateral tonic-clonic seizures. She developed myoclonic seizures at 20 months, followed by other generalized seizure types including tonic-clonic, tonic, and eyelid myoclonia with absence. She did not have febrile seizures. The patient’s EEG at seizure onset revealed multifocal sharp wave activity, and multiple repeat EEGs demonstrated slow dysrhythmic background, generalized and focal discharges, and strong photoconvulsive response. Ictal EEG demonstrated rhythmic generalized and bi-posterior quadrant spike and wave and polyspike and wave discharges which were time-locked with eyelid myoclonia (Fig 1a).

She failed clobazam, levetiracetam, lamotrigine, topiramate, and cannabinoid oil. She responded to valproic acid and clonazepam, but their up-titration was limited by side effects of weight gain and alopecia with the former and behavioural problems with the latter. Ethosuximide was added and a vagal nerve stimulator was inserted at age 9 with good effect. She currently has eyelid myoclonia with absence on a daily basis and tonic seizures once every 6 weeks.

The patient was conceived via in-vitro fertilization. She was born at 30-weeks gestation by C-section following a dizygotic twin pregnancy complicated by diet-controlled gestational diabetes. She did not require resuscitation at birth. Serial neuroimaging revealed periventricular leukomalacia and macrocephaly secondary to non-progressive ventriculomegaly (Fig 1b). Global developmental delay was observed before seizure onset. She was diagnosed with autism and mild to moderate intellectual disability on psychoeducational assessment at age 4. On family history, her father has generalized epilepsy which has been well-controlled since adolescence.

Neurological examination revealed macrocephaly, mild dysmorphism and diffuse hypotonia. Extensive metabolic screening was unremarkable and chromosomal microarray was normal. Targeted WES revealed a heterozygous *GABRA1* pathogenic variant (NM_000806: c.640C > T; p.R214C) which was confirmed by Sanger sequencing (Fig 1c).

### The R214C variant resulted in loss of function in GABA_A_Rs

**Fig. 2:**
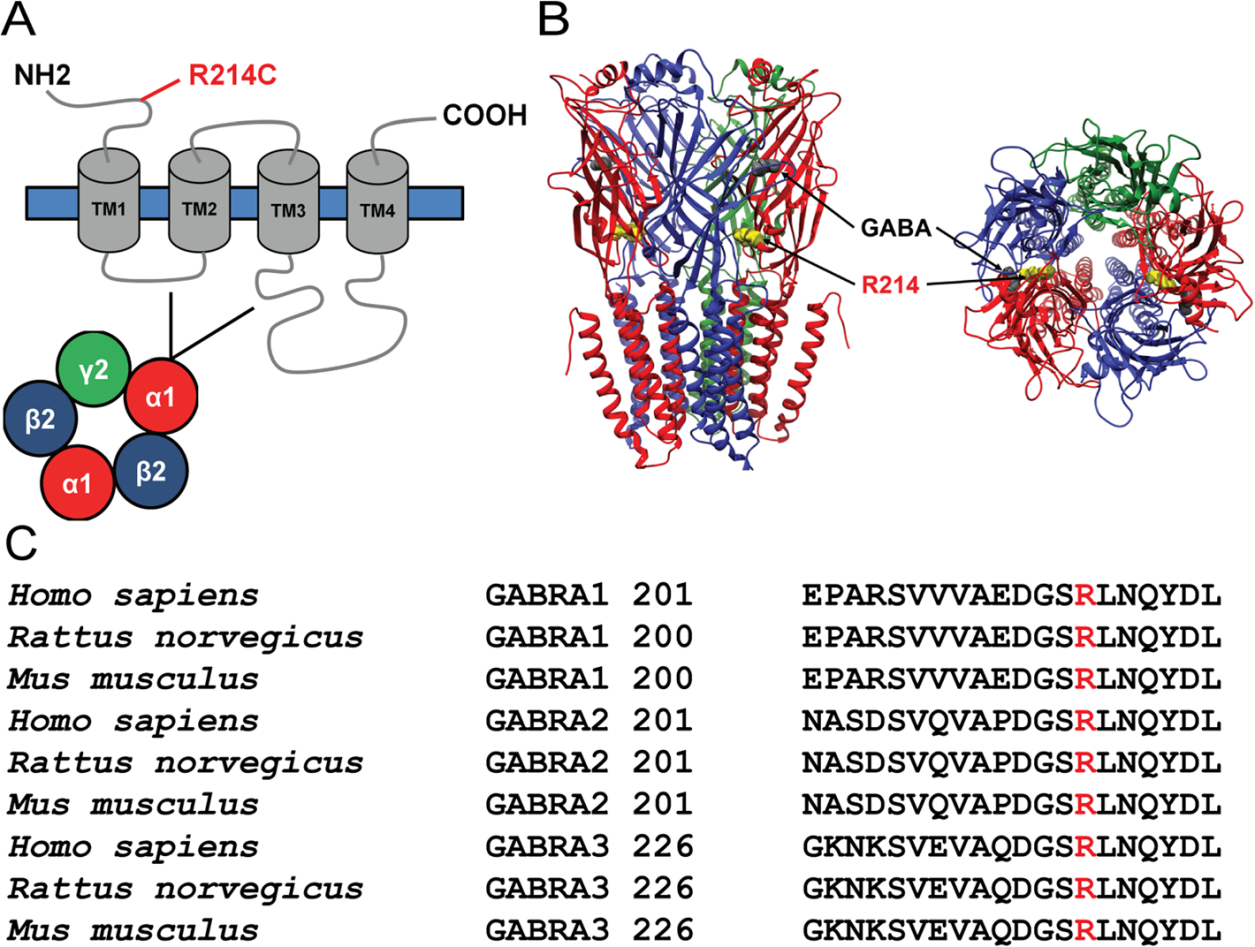
The residue of the GABRA1 (R214C) mutation is highly conserved across species. **a** Diagrammatic representation of the GABA_A_R α1 subunit. Mutation of the R214 residue is located in the extracellular N-terminal domain of the α1 subunit. **b** A three-dimensional structural model of GABA_A_Rs with the mutant site R214 indicated in yellow, and the GABA binding site, indicated in grey. Molecular graphics and analyses performed with UCSF Chimera, developed by the Resource for Biocomputing, Visualization, and Informatics at the University of California, San Francisco, with support from NIH P41-GM103311 [36]. **c** The R214 residue (highlighted in red in sequence alignments) is highly conserved among different species and across the different GABRA1–3 genes

The site of the R214C α1 variant is located in the extracellular N-terminal domain of the α1 subunit (Fig 2a), close to the GABA binding site (Fig 2b). The 214 residue is highly conserved amongst different species, including *Homo sapiens, Rattus norvegicus* and *Mus musculus*, and amongst different GABRA1–3 genes (Fig 2c), highlighting the potential importance of the residue. As this is a previously uncharacterized variant, we undertook functional studies to determine if it causally contributed to the patient’s pathological phenotype and, if so, to seek a better therapeutic strategy for the patient.

**Fig. 3:**
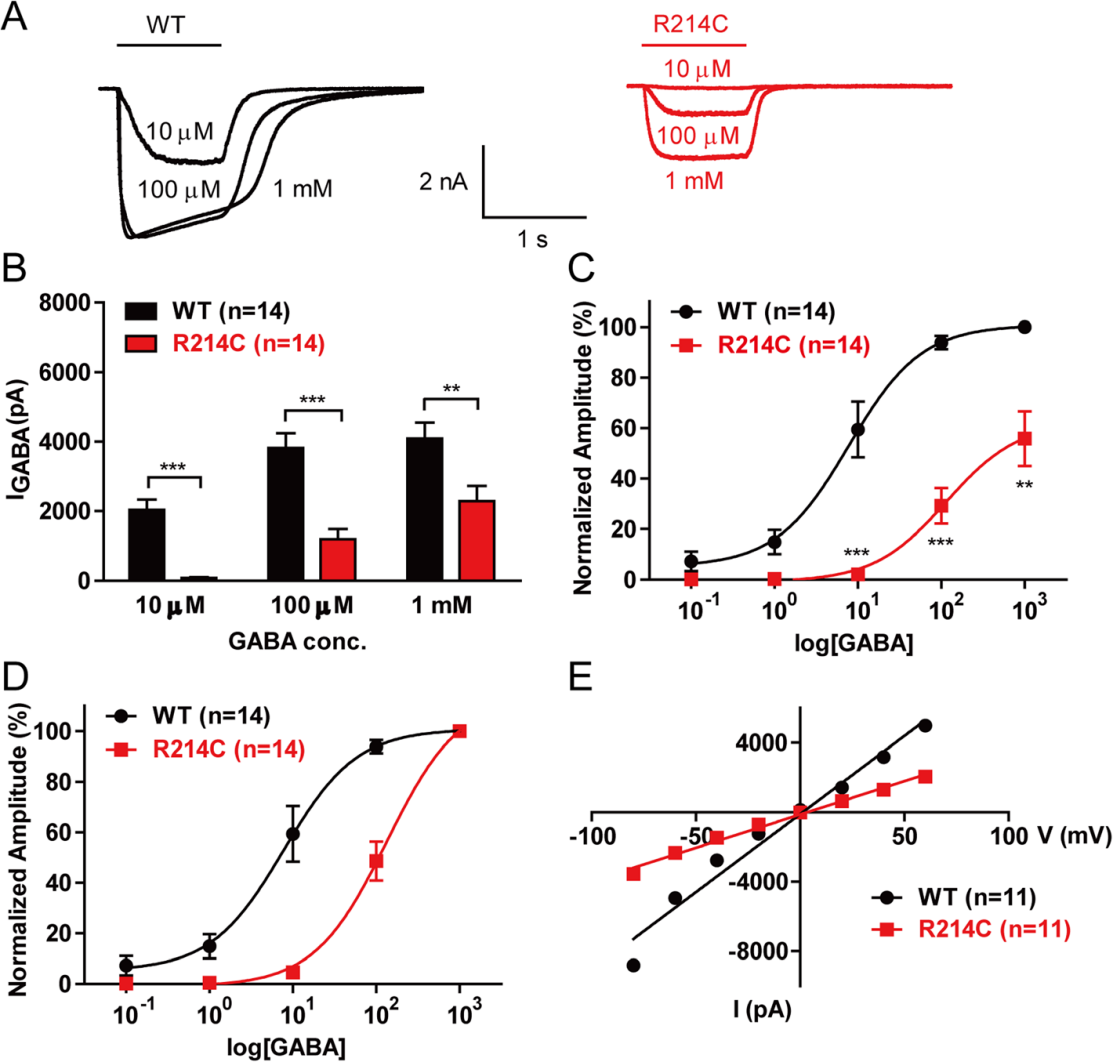
The R214C subunit mutation decreases GABA-evoked currents, without affecting chloride selectivity. **a** Representative GABA-evoked current traces from WT (Black) or R214C (Red) GABA_A_R expressing HEK293 cells, in response to fast applications of GABA at indicated concentrations. The cells were held at -60 mV and perfused with GABA at increasing concentrations. GABA application (1 s) is indicated as a black line at the top of the traces. **b** Quantification of the averaged peak current amplitudes from WT (*n* = 14) or R214C (*n* = 14) GABA_A_R expressing cells at increasing GABA concentrations (10 μM-1 mM). Statistical differences were determined using student’s *t*-test (**p < 0.01, ***p < 0.001). **c** Dose-response curves comparing GABA-evoked currents from R214C GABA_A_R expressing cells to WT GABA_A_R expressing cells. The peak current amplitude from R214C was normalized to the maximum response (1 mM GABA) from WT. Statistical differences was determined using student’s *t*-test (**p < 0.01, ***p < 0.001). **d** Dose-response curves for GABA-evoked currents from WT (*n* = 14) or R214C (n = 14) GABA_A_R expressing cells. The peak current amplitude at each GABA concentration for WT or R214C, was normalized to the maximum response (1 mM GABA) from each receptor, respectively. Data from (**c**) and (**d**) were fitted to the *Hill* eq. (**e**) Quantification of current-voltage (I/V) plots for GABA-evoked currents from of WT (*n* = 11) or R214C (*n* = 11) GABA_A_Rs. Cells were clamped from -80 mV to + 60 mV with a step of 20 mV. Data is represented as +SEM

To examine the effects of the R214C variant on GABA_A_R function, we measured GABA-evoked currents from WT α1β2γ2 (WT) and α1_R214C_β2γ2 (R214C) GABA_A_R expressing HEK293 cells using whole-cell voltage recordings at a holding membrane potential of -60 mV. Whole-cell currents were evoked by fast perfusion of GABA at different concentrations (10 μM-1 mM, 1 s). As shown in Fig 3, peak current amplitudes from R214C GABA_A_Rs were significantly reduced when compared to WT GABA_A_Rs at each GABA concentration (Fig 3a-b). We next examined the effect of the R214C mutation on GABA sensitivity by analyzing the dose-response relationship of the GABA-evoked currents at increasing doses of GABA (0.1 μM-1 mM, 1 s) from the same cell. When normalized against the maximum response of WT GABA_A_Rs (1 mM GABA), GABA-evoked currents from R214C GABA_A_Rs at all doses were significantly lower than that of WT from 10 μM-1 mM GABA (Fig 3c). In addition, when normalized to their own maximum responses at 1 mM GABA, we observed a rightward shift in the dose-response curve for R214C GABA_A_Rs (Fig 3d), and the EC_50_ for R214C GABA_A_Rs was significantly higher than that of WT GABA_A_Rs (R214C: 115.10 ± 1.70 μM; WT: 7.80 ± 1.36 μM).

To determine if the R214C variant affected R214C GABA_A_R anion selectivity, GABA-evoked peak current amplitudes of WT and R214C GABA_A_Rs were measured at membrane potentials from − 80 to + 60 mV with a step voltage of 20 mV. The mutation did not alter chloride selectivity as the reversal potential was near 0 mV in both WT and R214C GABA_A_Rs (Fig 3e). Therefore, our results demonstrate that the variant caused a reduction in both peak current amplitude and GABA sensitivity, without changing the chloride selectivity of the channel.

### The R214C variant resulted in reduced surface and total α1 subunit expression

We hypothesized that the R214C variant could impair receptor function through reducing receptor expression and function. GABA_A_R assembly and packaging in the ER is a tightly regulated process. The proper surface expression of the GABA_A_R requires the subunits to be assembled to form conformationally-mature pentameric GABA_A_Rs before exiting into the Golgi for traffic to the cell surface [29].

**Fig. 4:**
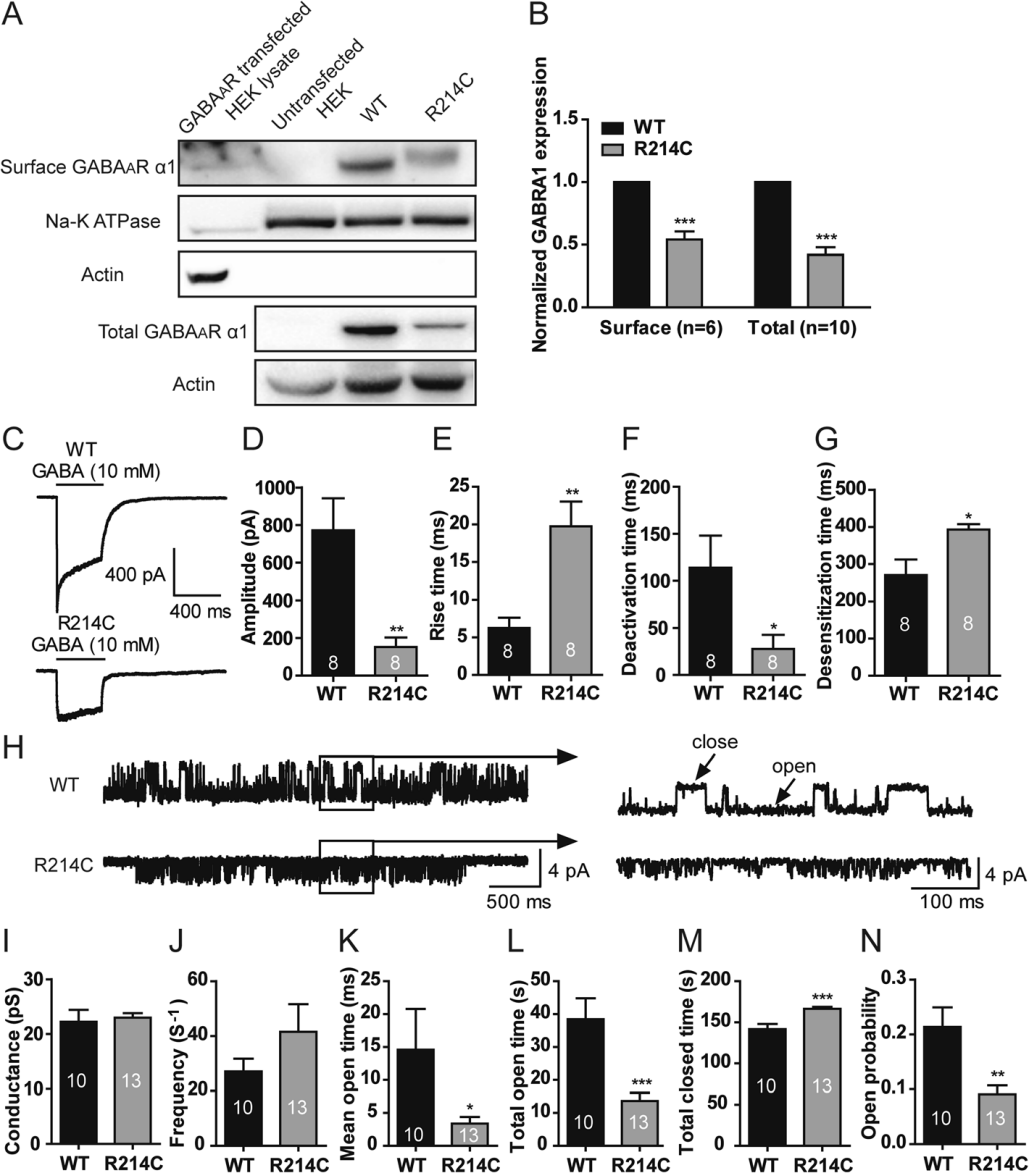
The R214C mutation resulted in reduced surface and total expression levels of the α1 subunit, and altered the kinetic and single channel properties of GABA_A_Rs. **a** Representative blots of biotinylation samples for surface receptor expression and cell lysates for total receptor expression from HEK293 cells expressing either WT or R214C GABA_A_Rs. **b** Quantification of surface α1 subunits normalized to Na^+^/K^+^ ATPase (*n* = 6), and total α1 subunits normalized to β-actin (*n* = 10). Statistical differences were determined using student’s *t*-test by comparing to expression levels of WT GABA_A_R expressing cells (***p < 0.001). **c** Representative traces of GABA currents recorded in excised macro-patch membrane under outside-out configuration from WT or R214C GABA_A_R expressing cells. Currents were evoked by rapidly perfusion of 10 mM GABA to the membrane patch for 400 ms. Quantification of averaged peak current amplitudes (**d**), 10–90% rise time (**e**), deactivation rate (**f**) and desensitization (**g**) in WT (*n* = 8) or R214C (*n* = 8) GABA_A_R expressing cells. **h** Representative single channel current traces recorded under cell-attached configuration with a pipette containing GABA (1 mM) at a holding potential of + 100 mV from cells expressing WT or R214C GABA_A_Rs. Quantified average of conductance (**i**), opening frequency (**j**), mean open time (**k**), total open time (**l**), total closed time (**m**), and open channel probability (**n**) of WT (*n* = 10) or R214C (*n* = 13) GABA_A_Rs. Statistical differences were determined using student’s *t*-test by comparing to WT GABA_A_R cells (*p < 0.05, **p < 0.01, ***p < 0.001)

To examine if the substantial decrease in GABA-evoked currents in the R214C GABA_A_Rs, could be, in part, a result of protein degradation due to misfolding of the mutated α1 subunit-containing receptor, we quantified the surface and total expression levels of α1 subunit in HEK cells expressing either WT or R214C GABA_A_Rs. We found that the variant reduced the surface and total levels of the α1 subunit in R214C GABA_A_Rs to 54.10 ± 6.50% and 41.95 ± 6.00% of the levels of WT GABA_A_Rs, respectively (Fig 4a, b). These results are consistent with the conjecture that R214C GABA_A_Rs are misfolded and degraded intracellularly, thereby preventing their export to the cell surface.

### The R214C variant altered GABA current kinetics and GABA_A_Rs single channel properties

To determine if the variant directly impacted channel gating properties, we examined the activation and deactivation rate as well as the desensitization of WT and R214C GABA_A_Rs on excised membrane patches under the configuration of outside-out patch-recordings currents. Currents recorded from such macropatches provide much better temporal resolution for analyzing channel gating properties, including kinetics. We applied brief pulses of a saturating concentration of GABA (10 mM, 400 ms) to fully activate the receptor channels on the membrane patches excised from HEK cells expressing WT or R214C GABA_A_Rs (Fig 4c).

Consistent with the results observed under whole-cell recording shown in Fig 3a and b, we found that the peak currents of R214C GABA_A_Rs (− 151.96 ± 50.40pA) were significantly smaller than that of WT GABA_A_Rs (− 772.14 ± 169.64pA) (Fig 4d). In addition, R214C GABA_A_Rs showed significantly slower activation (10–90% rise time), with an average rate of 19.75 ± 3.29 ms, as compared to that of WT GABA_A_Rs (6.25 ± 1.39 ms) (Fig 4e). The R214C GABA_A_Rs also showed faster deactivation rate (27.73 ± 14.95 ms) as compared to WT (113.86 ± 34.30 ms) (Fig 4f) and slower desensitization (392.99 ± 14.25 ms) as compared to WT (270.52 ± 41.65 ms) (Fig 4g).

These results strongly suggest that altered channel gating properties may contribute to the reduced function of R214C GABA_A_Rs. To further determine the effects of the R214C variant at the single channel level, we performed single channel recordings using cell-attached single channel currents of WT or R214C GABA_A_Rs induced by GABA (1 mM) contained in the patch pipette. Single channel currents displayed channel openings with complex bursting patterns (Fig 4h). There were no significant differences in the levels of single conductance between WT (22.24 ± 2.18pS) and R214C receptors (22.99 ± 0.82pS) (Fig 4i). However, R214C GABA_A_Rs showed significantly lower open probability (WT: 0.21 ± 0.04; R214C: 0.09 ± 0.02, Fig 4n) as the result of reduced mean open time (WT: 14.59 ± 6.20 ms; R214C: 3.38 ± 1.00 ms, Fig 4k) and total open time (WT: 38.43 ± 6.40s; R214C: 13.63 ± 2.46 s, Fig 4l), and increased total closed time (WT: 141.57 ± 6.40s; R214C: 166.37 ± 2.46 s, Fig 4m). While there was a noticeable increase in opening frequency of R214C GABA_A_Rs over WT counterparts (WT: 27.05 ± 4.67 Hz; R214C: 41.56 ± 10.08 Hz, Fig 4j), it was not statistically significant. These results demonstrate that the R214C variant reduces GABA_A_R function primarily through decreasing its channel open probability, a property that is thought to be largely dependent on agonist binding affinity.

### GABA-evoked currents in R214C GABA_A_Rs were partially rescued by diazepam and insulin

As described above, we demonstrated that the R214C variant causes loss-of-function through reduction in surface receptor expression and impairment of receptor functioning. We examined if these functional deficits could be rescued with either diazepam, a positive allosteric GABA_A_R modulator that has previously been shown to increase channel opening and conductance, [31, 43, 44] or insulin, which has been previously shown to increase the number of surface GABA_A_Rs by facilitating receptor translocation from intracellular compartments to the plasma membrane [32].

**Fig. 5:**
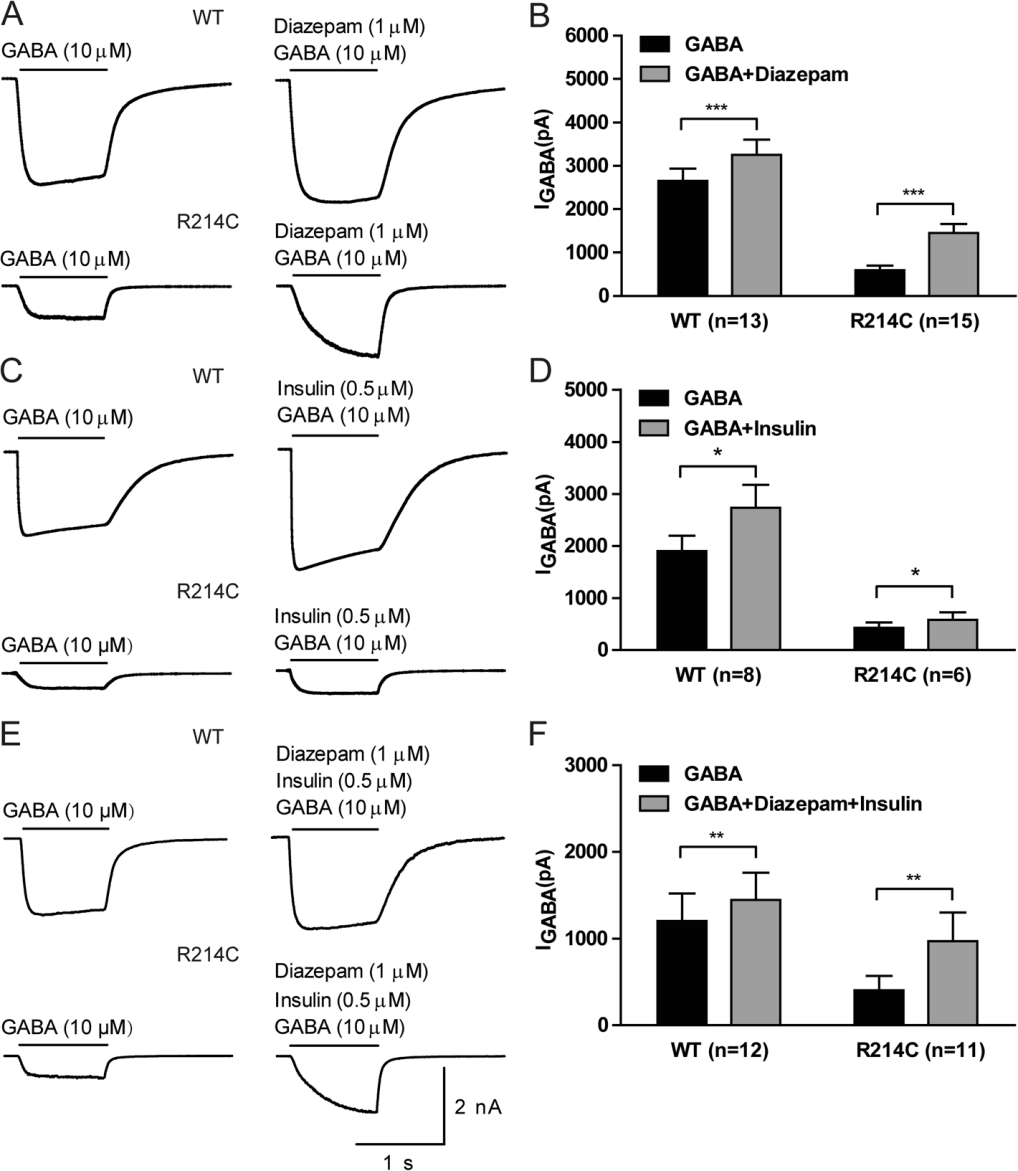
Insulin or diazepam or their combination could only partially rescue the functional deficits of R214C GABA_A_Rs. **a** Representative traces of GABA (10 μM, 1 s)-evoked currents from WT or R214C GABA_A_R expressing cells, with or without rapid diazepam application (1 μM, 1 s). **b** Quantification of averaged peak current amplitudes recorded from cells expressing WT (n = 13) or R214C (*n* = 15) GABA_A_Rs before and after exposure to diazepam. **c** Representative traces of GABA-evoked currents from WT or R214C GABA_A_R expressing cells, with or without insulin (0.5 μM, 10 min) treatment. Cells were first serum starved for 2 h prior to recording, and GABA currents were then evoked before and after treatment of insulin for 10 min. **d** Quantification of averaged peak current amplitudes recorded from cells expressing WT (*n* = 8) or R214C (*n* = 6) GABA_A_Rs before and after insulin treatment. **e** Representative traces of GABA-evoked currents from WT or R214C GABA_A_R expressing cells before and after insulin and diazepam co-treatment. Cells were first serum starved for 2 h before recording an initial GABA-evoked current (10 μM GABA, 1 s). The same cell was then perfused with insulin (0.5 μM, 10 min) in the recording chamber, and a second GABA-evoked current (1 μM diazepam, 0.5 μM insulin, 10 μM GABA, 1 s) was recorded thereafter. **f** Quantification of averaged peak current amplitudes recorded from cells expressing WT (*n* = 12) or R214C (*n* = 11) GABA_A_Rs before and after insulin and diazepam co-treatment. Statistical differences were determined using paired *t*-test (*p < 0.05, **p < 0.01, ***p < 0.001). Data is represented as +SEM

We recorded GABA-evoked currents (10 μM, 1 s) from WT and R214C GABA_A_R expressing cells with and without diazepam (1 μM, 1 s). 10 μM of GABA was used, as it was the concentration that exerted a sub-maximal response in both receptors. As previously reported, diazepam was able to enhance both WT and R214C GABA_A_R currents (Fig 5a, b). However, GABA currents of R214C GABA_A_Rs with diazepam only reached 54.49% of WT receptors in the absence of diazepam.

We have previously shown that insulin potentiates GABA-evoked current amplitudes by increasing postsynaptic GABA_A_R expression [32]. This process was reported to be dependent on the activation of phosphoinositide 3-kinase (PI3-K)-dependent Akt phosphorylation of GABA_A_R β subunits [45, 46]. We therefore investigated if insulin would also be able to, at least in part, restore the function of the R214C mutant receptor by increasing its surface expression. After serum-starving the cells (to remove residual insulin from the culture media) in ECS for 2 h, we first recorded an initial GABA-evoked current (10 μM GABA, 1 s) in the absence of exogenous insulin, and then the currents (0.5 μM insulin, 10 μM GABA, 1 s) following perfusion with insulin (0.5 μM, 10 min) in the recording chamber (Fig 5c).

Insulin produced a 30% increase in the GABA-evoked currents in cells expressing WT receptors (GABA: − 1899.65 ± 295.43pA; GABA+Insulin: − 2729.24 ± 444.36pA; Fig 5c and d) and a 35.73% increase in GABA currents in the mutant receptor expressing cells (GABA: − 426.96 ± 105.83pA; GABA+Insulin: − 579.53 ± 147.61pA; Fig 5c and d). However, the currents through the mutant receptor after insulin treatment were much smaller than currents through the WT receptor without insulin treatment (Fig 5c and d). This ineffectiveness of insulin rescue on the mutant receptors may not be surprising given that the variant significantly reduced total receptor expression, including in intracellular compartments upon which insulin acts (Fig 4a and b).

As diazepam and insulin act independently to partially increase the function of R214C mutant receptors, we determined if co-application of diazepam and insulin could synergistically increase GABA-evoked currents in R214C GABA_A_Rs. A combination of 1 μM diazepam and 0.5 μM insulin produced a pronounced enhancement of the currents in R214C expressing cells, increasing from − 403.78 ± 168.22pA to − 972.13 ± 327.42pA, which is equivalent to 80.97% of the currents through WT receptors in the absence of diazepam and insulin (Fig 5e, f). Thus, the combination of diazepam and insulin synergistically rescued the function of the mutant receptor.

### Treatment with verapamil rescued deficient GABA-evoked currents in R214C GABA_A_Rs without increasing surface GABA_A_R expression

**Fig. 6:**
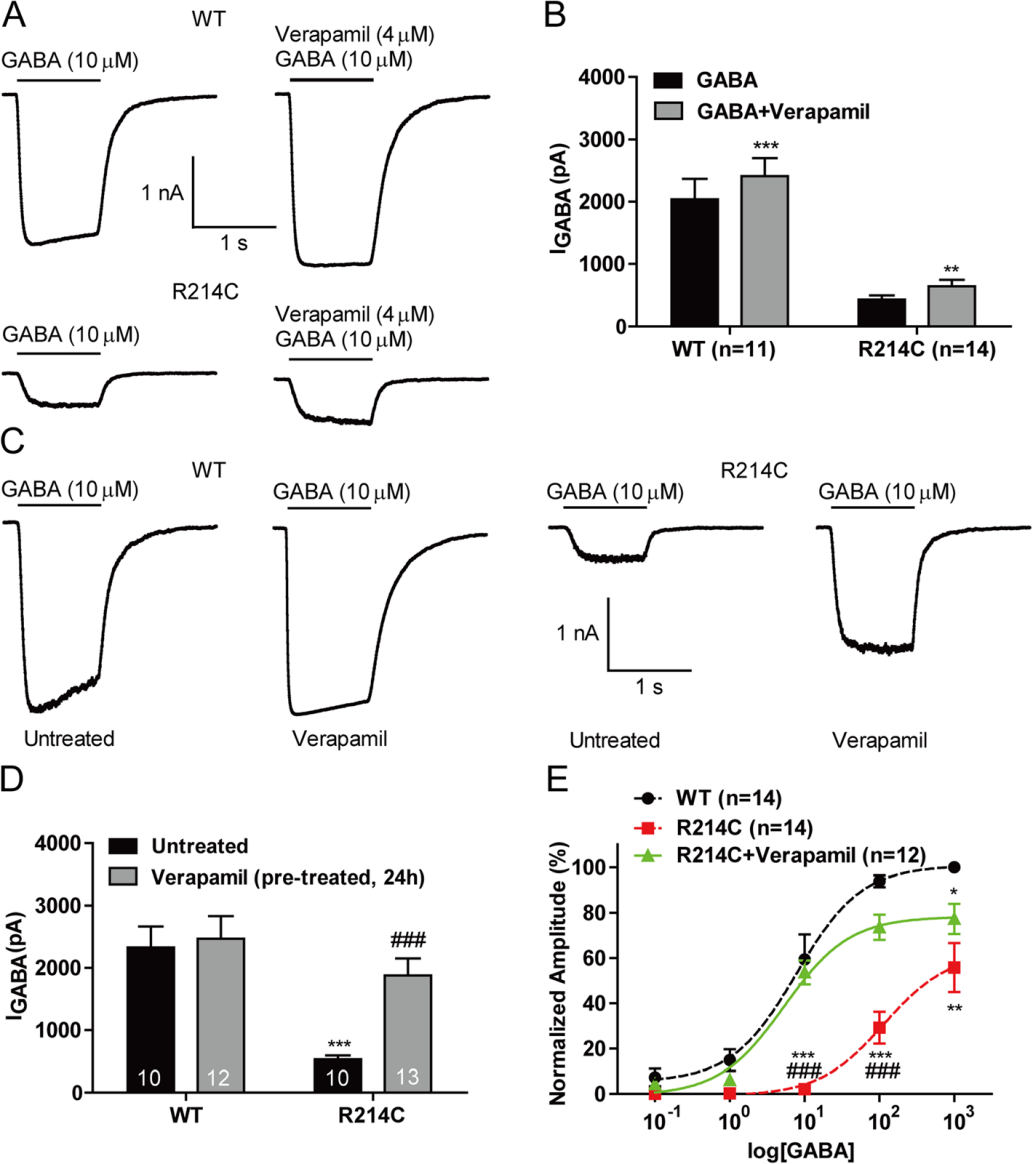
Chronic, but not acute, verapamil treatment fully restored GABA-evoked chloride currents in R214C GABA_A_R expressing cells. **a** Representative traces of GABA-evoked currents from cells expressing WT or R214C GABA_A_Rs without and with verapamil (4 μM, 1 s co-applied with GABA). **b** Quantification of averaged peak current amplitudes recorded from cells expressing WT (*n* = 11) or R214C (n = 14) GABA_A_Rs without and or with verapamil. Statistical differences were determined using paired *t*-test (**p < 0.01, ***p < 0.001). (**c**) Representative traces of GABA-evoked currents from WT or R214C GABA_A_R expressing cells that were without (untreated) and pre-incubated with 4 μM verapamil for 24 h (Verapamil). **d** Quantification of averaged peak current amplitudes recorded from WT (*n* = 10, 12) or R214C (*n* = 10, 13) GABA_A_Rs that were untreated or treated with verapamil (4 μM, 24 h), respectively. Statistical differences were determined using two way ANOVA followed by post hoc student’s *t*-test by comparing GABA-evoked currents of untreated WT GABA_A_R expressing cells (***p < 0.001), or untreated R214C GABA_A_R expressing cells (*###p < 0.001*). **e** Dose-response curve for GABA-evoked currents from R214C GABA_A_R expressing cells pre-treated with 4 μM verapamil for 24 h (*n* = 12). The dose curve for verapamil treated (4 μM, 24 h) R214C GABA_A_Rs was replotted against the dose response curve in Fig. 3. The peak current amplitudes at each GABA concentrations were normalized to the maximum responses from WT GABA_A_R (1 mM GABA). Statistical differences were determined using student’s *t*-test by comparing to the GABA-evoked currents from WT (*p < 0.05, **p < 0.01, ***p < 0.001), or R214C (*###p < 0.001*), at the corresponding GABA concentration. Data is represented as +SEM

A recent study reported that verapamil, a L-type calcium channel blocker, fully rescued the function of GABA_A_Rs with a D219N α1 variant by increasing receptor assembly at the ER and enhancing trafficking to the plasma membrane [33]. As D219N is very close to the R214C variant in the same subunit, we tested if verapamil could also rescue the functional deficits in R214C GABA_A_Rs. We first examined if there were any acute effects verapamil on GABA_A_R function by recording GABA-evoked currents in both WT and R214C receptors expressed in HEK cells. Acute application of verapamil (4 μM, 1 s) resulted in a small but significant increase in GABA-evoked currents in both WT and R214C GABA_A_Rs (Fig 6a, b). As this effect is acute, it is unlikely a result of improved receptor assembly and/or membrane trafficking. Instead, it suggests an acute effect of verapamil on channel gating. However, this channel-gating effect would be too small to restore the function of the mutant receptor to that of WT.

We then tested if chronic treatment of verapamil could produce greater levels of rescue through improving receptor assembly and/or plasma membrane expression. In contrast to the acute treatment, we found that a chronic verapamil treatment (4 μM, 24 h) of cells expressing the R214C significantly increased GABA-evoked currents, fully restoring it to a level that is not statistically different from the currents of untreated WT receptors (Fig 6d). More importantly, this dramatic potentiation induced by chronic verapamil treatment was only specifically observed for the mutant, but not WT, GABA_A_Rs (WT: − 2328.01 ± 335.43pA, WT + Verapamil: − 2467.36 ± 364.01pA; R214C: − 533.27 ± 62.33pA, R214C + Verapamil: − 1877.71 ± 272.46pA, Fig 6c, d, Additional file 1: Figure S1). Detailed GABA dose-response analysis revealed that chronic verapamil treatment caused a rightward shift in the dose-response curve of the currents of R214C GABA_A_Rs; and interestingly, full restoration of the function of mutant receptors was only observed in the range of 10 μM–100 μM of GABA (Fig 6e). At higher mM concentrations of GABA, verapamil substantially increased GABA currents through R214C receptors but failed to restore the currents to the level of WT GABA_A_R. These results suggest that chronic verapamil treatment may have a preferential effect on the receptor under unsaturated conditions (Fig 6e).

**Fig. 7:**
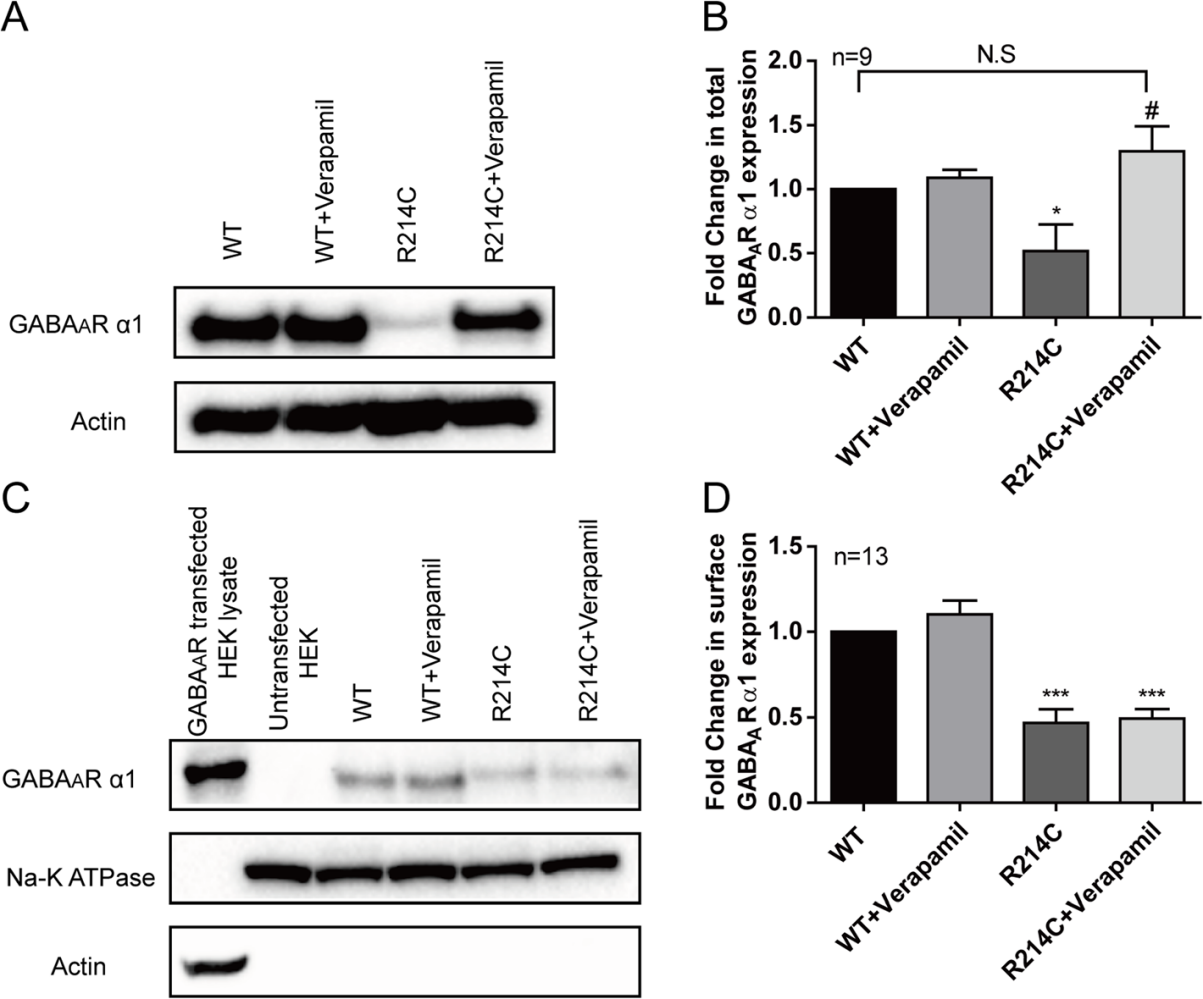
Verapamil treatment increased total, but not surface expression levels of α1 subunit in R214C GABA_A_R expressing cells. **a** Representative whole cell lysate blots from WT and R214C GABA_A_R expressing cells. **b** Quantification of total expression levels of α1 subunit in untreated or verapamil treated (4 μM, 24 h) WT and R214C GABA_A_R expressing cells (*n* = 9). **c** Representative surface expression blots from WT and R214C GABA_A_R expressing cells. **d** Quantification of surface expression levels of α1 subunits, normalized to Na^+^/K^+^ ATPase expression levels, and represented as a fold change against untreated WT GABA_A_R expressing cells (*n* = 13). Statistical differences were determined using two way ANOVA followed by post hoc student’s *t*-test by comparing to expression levels of untreated WT expressing cells (*p < 0.05, ***p < 0.001), or untreated R214C expressing cells (*#p < 0.05*). Data is represented as +SEM

To determine if the functional rescue of R214C GABA_A_Rs by chronic verapamil treatment was indeed a result of increased surface expression of mutant GABA_A_Rs, as reported elsewhere, [33] we performed biochemical evaluation of the changes in R214C receptor expression on the plasma membrane surface with surface biotinylation and total expression in the cells with immunoblotting. Verapamil treatment did not alter either total (Fig 7a and b) or surface (Fig 7c and d) WT GABA_A_Rs. Verapamil increased total α1 subunit expression of R214C GABA_A_Rs to WT level (Fig 7a and b) but failed to increase their expression on the plasma membrane surface (Fig 7c, d), which suggests that the full rescue of receptor function by chronic verapamil treatment is not mediated by increasing the number of functional receptors.

### Verapamil rescued deficiency in chloride currents by altering channel gating properties of R214C GABA_A_Rs

**Fig. 8:**
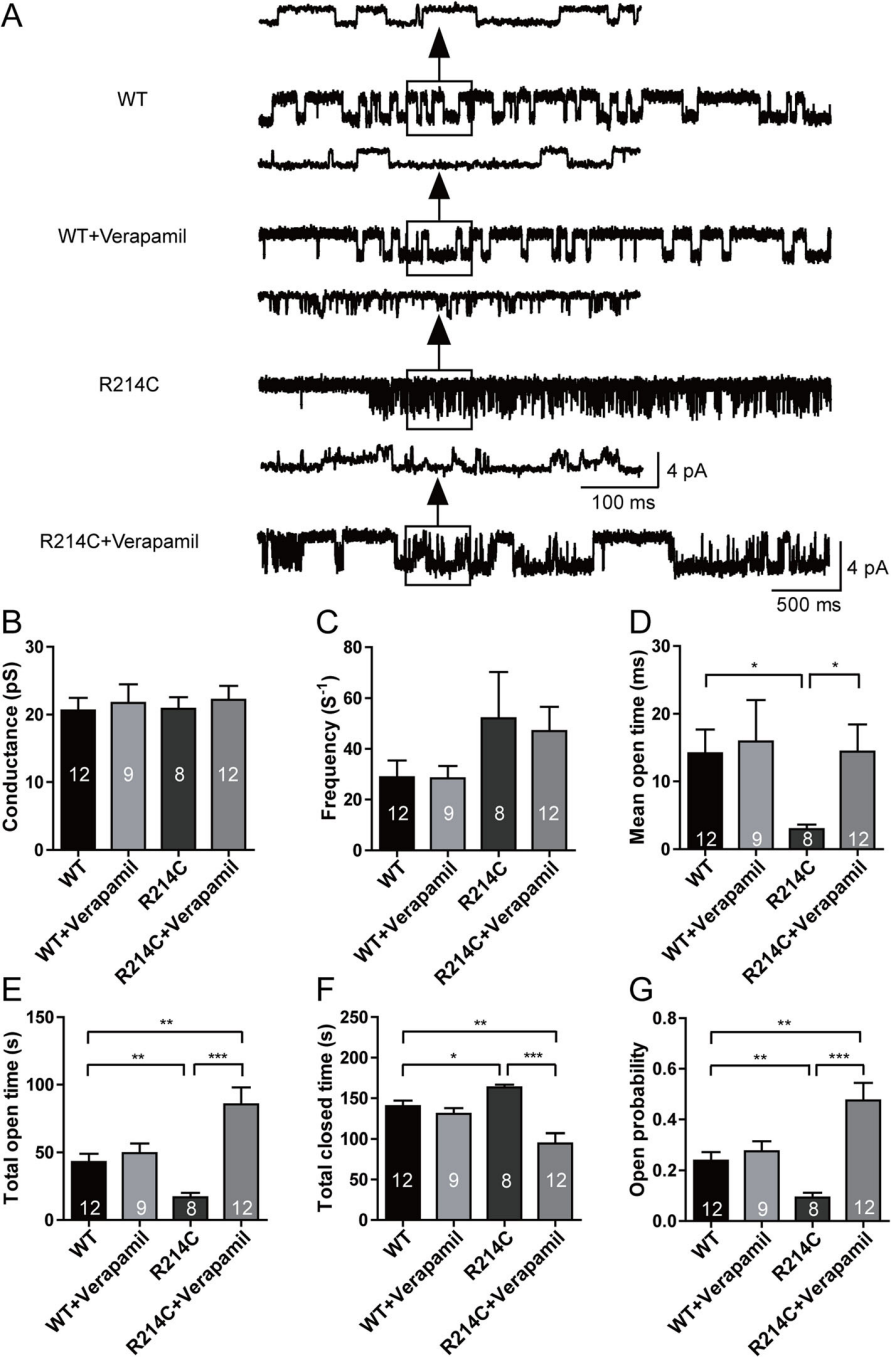
Verapamil increased single channel open time and open channel probability in R214C GABA_A_Rs. **a** Representative single channel current traces from untreated or verapamil treated (4 μM, 24 h) WT (*n* = 9, 12) and R214C (*n* = 8, 12) GABA_A_Rs, respectively. WT and R214C GABA_A_R expressing cells were recorded under cell-attached configuration with a recording pipette containing GABA (1 mM) at a holding potential of + 100 mV, and the single channel conductance (**b**), frequency (**c**), mean open time (**d**), total open time (**e**), total closed time (**f**) and channel open probability (**g**) were quantified. Statistical differences were determined using two way ANOVA followed by post hoc student’s *t*-test by comparing untreated WT, or untreated R214C (*p < 0.05, **p < 0.01, ***p < 0.001). Data is represented as +SEM

We explored the possibility that verapamil treatment restored function of the mutant receptor through altering its channel gating properties by performing single-channel electrophysiological recordings of GABA currents under the on-cell attached configuration. As compared to untreated WT and R214C GABA_A_Rs, verapamil treatment dramatically increased total open time (WT: 42.93 ± 6.13 s; R214C: 16.79 ± 3.29 s; R214C + Verapamil: 85.50 ± 12.44 s, Fig 8e) and hence open channel probability (WT: 0.24 ± 0.03; R214C: 0.09 ± 0.02; R214C + Verapamil: 0.48 ± 0.07, Fig 8g). Concurrently, treatment decreased the total closed time (WT: 140.01 ± 7.01 s; R214C: 163.21 ± 3.29 s; R214C + Verapamil: 94.50 ± 12.44 s, Fig 8f). While the mean open time of untreated and verapamil treated WT and R214C GABA_A_Rs were not statistically significant by two way ANOVA, this could be due to the high variability among each channel open time of the GABA_A_Rs. Nonetheless, mean channel open times were statistically significant when comparing between untreated WT and R214C GABA_A_Rs, and untreated and verapamil treated R214C GABA_A_Rs (WT: 14.17 ± 3.52 ms; R214C: 2.94 ± 0.68 ms R214C + Verapamil: 14.40 ± 4.04 ms, Fig 8d). These results strongly suggest that chronic verapamil treatment restores the function of the R214C mutant receptor primarily by enhancing channel activity, rather than by increasing receptor expression on the cell surface.

## Discussion

We identified a de novo *GABRA1* pathogenic variant (R214C) in a patient with EE, treatment-resistant epilepsy, intellectual disability and autism. Her clinical presentation falls on the severe end of the phenotypic spectrum and she shares features that are similar to previously described patients with *GABRA1* variants including early-onset EE, the presence of myoclonic and generalized tonic seizures as well as photoparoxysmal response on EEG [13].

The R214C residue of the α1 GABA_A_R subunit is located in the extracellular N-terminus domain close to the GABA binding site (Fig. 2b), and is highly conserved amongst different species, including *Homo sapiens*, *Rattus norvegicus* and *Mus musculus*, and amongst different *GABRA1–3* genes (Fig. 2c), suggesting the potential importance of this residue.

Consistent with this postulation, several potentially pathogenic variants at this site have been reported. In addition to the patient presented in this study, the same de novo variant has been identified through clinical testing in two other patients listed in ClinVar. One of them presented with intractable seizures (SCV000321687.6) while no clinical information was provided for the second patient (SCV000804975.1). Variants at the same protein location but with a different amino acid change have also been identified in two patients, both carrying a c.641G > A; p.R214H varian t[13]. Similar to our patient, they had severe phenotypes. One had a Dravet Syndrome-like presentation. The other was diagnosed with EE and had intractable seizures, developmental delay and generalized sharp waves with paroxysmal activity on EEG. However, unlike our patient, she presented with prolonged febrile seizures at 15 months of age and her MRI head was normal.

Thus, the present work together with these previous studies, strongly suggests that the GABRA1 R214 site is functionally critical and can be affected by pathogenic mutations. This highlights the importance of functional characterization of the R214C mutation in order to determine its pathophysiology. To our knowledge, functional studies of the R214C mutation have not been previously performed or published.

In this study, we characterized the effect of the R214C mutation on GABA_A_Rs. Our analysis provided compelling evidence that epileptogenesis of this novel variant was a result of decreased inhibitory tone, as evidenced by a significant reduction of GABA-evoked whole-cell currents and an increase of the GABA EC_50_ value. These results demonstrate that the R214C variant produced dysfunctional GABA_A_Rs that could not provide the sufficient level of neuronal inhibition required for normal functioning of neurons and developmental maturation of neural circuits. Furthermore, our results are consistent with previous studies demonstrating that GABRA1 variants cause loss-of-function of GABA_A_Rs [1, 7, 13, 18].

Several underlying mechanisms may be involved in a severe loss-of-function of mutant GABA_A_Rs, including 1) reducing surface receptors by altering receptor expression, assembly and trafficking; 2) reducing GABA-sensitivity by changing agonist-binding interface; 3) impairing channel opening by affecting receptor conformation-change efficiency [6, 8, 12, 19].

N-terminal sequences are important for expression, assembly and intracellular trafficking of GABA_A_Rs [47]. Our surface biotinylation and western blotting data showed a significant reduction in surface and total R214C GABA_A_Rs. A loss in total R214C GABA_A_R expression suggests that the mutant GABRA1 protein was either retained and degraded intracellularly, rather than trafficked to the plasma membrane surface, or that the mutation inhibited folding and assembly to form functional pentameric GABA_A_Rs [22, 29, 47]. Further studies are necessary to determine whether this mutation altered protein synthesis, folding, degradation, subunit assembly or receptor exocytosis.

The results from our electrophysiological recordings suggested that the reduction of total and surface GABA_A_Rs could not fully account for the significant decrease in GABA-evoked whole-cell currents. Our single channel data demonstrated that alteration of channel gating properties, also contributed significantly to the functional consequences of this variant. Kinetic changes in R214C GABA_A_R, including prolonged activation, accelerated deactivation and slowed desensitization, strongly suggest a decreased microscopic affinity of the mutated receptor for the agonist. Furthermore, the close proximity of the mutation site to the GABA binding site suggests that the variant may affect the ligand-binding coupling mechanism. Specifically, the changes to the GABA binding pocket by this variant may severely affect GABA binding/unbinding steps, which influences the transitions between open, closed, and desensitized states that are the major determinants of IPSC duration [48].

In addition, our single channel data analysis revealed that the R214C variant decreased open probability as well as mean and total channel open time without changing channel conductance. This further demonstrates that the mechanism underlying R214C GABA_A_R impairment was at least in part mediated through the alteration of GABA-binding affinity, thereby impacting receptor channel gating. While the detailed mechanisms by which the R214C variant exerts its impacts on channel gating remain unclear, alteration in charge strength due to the conversion of the positively-charged arginine residue to the neutral cysteine residue may play an important role.

The previously identified R214H mutation [13] may exert its impact on GABA_A_R function through a similar mechanism as the R214C mutation. Theoretically, at physiological pH, the charge change from arginine to cysteine (positive charge to neutral for R214C) is larger than the charge change from arginine to histidine (positive to less positive charge for R214H). As a result, we would expect that the functional impact of the R214C mutation on GABA_A_Rs should be greater than that of the R214H mutation. In supporting this conjecture, we observed that the R214C mutation resulted in greater GABA_A_R impairment than the R214H mutation [13].

Thus, using a combination of biochemical and electrophysiological characterization, our study provides convincing evidence that the loss-of-function phenotype of the R214C GABA_A_Rs is a result of reduced receptor number on the plasma surface and impaired receptor channel gating. This understanding of the variant’s underlying pathophysiologic mechanisms helped guide our search for therapeutic strategies to restore the function of mutant receptors. To this end, we tested the effects of diazepam, a positive allosteric GABA_A_R channel gating modulator, [31, 43, 44] and insulin, which we have previously shown to increase the surface expression of GABA_A_Rs [32].

We found that diazepam increased levels of GABA-evoked currents to 54.5% of WT level. This is consistent with our clinical observation of partial response in our patient’s seizures to clonazepam (benzodiazepine), though its use was limited by sedation. Insulin potentiated GABA-evoked currents of mutant R214C GABA_A_R to only 30.5% of WT level. When diazepam and insulin were applied together with diazepam, they rescued GABA-evoked currents of mutant R214C GABA_A_Rs to 80.9% of WT GABA_A_Rs. This suggests that diazepam and insulin work synergistically and could theoretically be a more effective strategy for patients with the R214C variant, but there are practical obstacles to using insulin as an anti-seizure medication given its potent adverse effect of hypoglycemia.

We then examined the effects of verapamil, a L-type calcium channel blocker, which is primarily used in the treatment of hypertension and as migraine prophylaxis. Verapamil has previously been trialed in patients with treatment-resistant epilepsy due to its property as a P-glycoprotein inhibitor and has been found to be well-tolerated but with mixed results on efficacy [49, 50]. Importantly, verapamil has recently been reported to increase surface expression of GABA_A_Rs, thereby fully restoring GABA-evoked currents in D219N GABA_A_Rs [33].

We observed that acute verapamil application resulted in a small potentiation of GABA-evoked currents in both WT and R214C GABA_A_Rs, suggesting that verapamil itself could be a positive allosteric modulator of GABA_A_Rs likely through improving channel gating. Chronic treatment with verapamil incubation for a period of 24 h fully rescued functional impairments on GABA_A_Rs caused by R214C variant, increasing the GABA-evoked currents to levels comparable to that of WT receptor.

Surprisingly, chronic verapamil treatment did not affect the GABA currents of WT GABA_A_Rs, indicating that chronic verapamil appears to have a specific effect on restoring the function of R214C mutant receptors. The mechanisms underlying variant-specific modulation of verapamil remain unclear. One potential explanation could be that the functional effects of verapamil are primarily mediated by improving GABA_A_R folding and maturation, processes which are compromised with the R214C variant, but less so with WT receptors.

Chronic verapamil treatment failed to increase R214C GABA_A_R expression on the plasma surface despite significantly increasing total receptor protein levels. This suggests that R214C may affect the stability of GABA_A_Rs and plasma membrane trafficking of GABA_A_Rs via different mechanisms, thus only exerting its effects on the former and not the latter. In addition, it indicates that verapamil’s ability to fully rescue GABA-evoked currents in R214C GABA_A_Rs to WT level is not due to increased receptor expression.

Our results are in contrast to that of a previous study, which showed that verapamil increased both total and surface expression of D219N of α1 GABA_A_Rs [33]. However, the results of this study were challenged by another recent study on the same variant which showed that α1 D219N GABA_A_Rs were actually less retained in the ER, having a similar pan-cadherin and α1 expression as WT GABA_A_Rs [15]. The absolute surface expression levels of D219N GABA_A_Rs seemed also comparable to that of WT GABA_A_Rs [15]. Therefore, whether the increase in GABA-evoked currents in D219N GABA_A_Rs treated with verapamil is due to an increase in surface trafficking may require additional validation.

Our data from single channel recordings suggests that verapamil exerts its effects through enhanced channel gating. Following chronic verapamil treatments, each R214C GABA_A_R channel opened for a much longer time, yielding a higher open probability, as compared to both untreated R214C and WT GABA_A_Rs. This increased duration for GABA currents to flux may have compensated for the reduced surface expression of R214C GABA_A_Rs, thereby attaining full rescue of GABA-evoked currents without the need of increasing surface receptor expression.

Full rescue was obtained with GABA concentrations ranging from 10 to 100 μM. This implies that verapamil not only targets synaptic GABA_A_Rs that are usually activated by GABA released from the presynaptic terminal, but also extra-synaptic GABA_A_Rs that are constantly activated by low concentrations of extracellular ambient GABA in the CNS and have an important role in reducing the contribution of each EPSP in reaching the threshold for action potential firing [51, 52]. This process is crucial in preventing uncontrolled action potential firing, which is a pathological hallmark of epileptogenesis [53–55].

In conclusion, our detailed characterization of the α1R214C variant’s functional impact on GABA_A_Rs provides strong evidence that it has a causative role in the pathological phenotype of our patient with EE. We demonstrated that a combination of enhancement of channel activity with benzodiazepines and upregulation of surface receptor expression with insulin largely restored function of mutant receptors. Our study also established that verapamil fully rescues mutant receptor function to wild type level and is a potentially effective therapeutic option for treatment of α1-related EEs. The precise mechanisms through which these drugs, particularly verapamil, improve the function of R214C GABA_A_Rs remains to be further studied.

Given that all of these drugs are currently in clinical use, our work may have an immediate impact on patient management. Ultimately, our study highlights the clinical importance of performing detailed functional and pharmacological characterizations of GABA_A_R variants in order to tailor the management of patients with genetic EEs through precision medicine.

## Supplementary information

**Additional file 1: Figure S1.** Verapamil induced maximum GABA-evoked chloride currents in R214C at 4 μM.

## Data Availability

Data supporting our findings are found within the manuscript and in the additional files.

## Abbreviations

EE: Epileptic encephalopathy
GABA: Gamma-aminobutyric acid
GABA_A_R: GABA A receptor
HEK: Human embryonic kidney
WES: Whole-exome sequencing
WT: Wild-type
