# Correction to: Pathophysiology of and therapeutic options for a *GABRA1* variant linked to epileptic encephalopathy

**DOI:** 10.1186/s13041-020-00593-6

**Published:** 2020-03-27

**Authors:** Yun-Fei Bai, Michelle Chiu, Elizabeth S. Chan, Peter Axerio-Cilies, Jie Lu, Linda Huh, Mary B. Connolly, Ilaria Guella, Matthew J. Farrer, Zhi-Qing David Xu, Lidong Liu, Michelle Demos, Yu Tian Wang

**Affiliations:** 1 Djavad Mowafaghian Centre for Brain Health and Department of Medicine, University of British Columbia, Vancouver, Canada; 2 Department of Neurobiology, Beijing Key Laboratory of Neural Regeneration and Repair, Beijing Laboratory of Brain Disorders (Ministry of Science and Technology), Beijing Institute for Brain Disorders, Capital Medical University, Beijing, China; 3 Division of Neurology, Department of Paediatrics, BC Children’s Hospital, University of British Columbia, Vancouver, Canada; 4 Centre for Applied Neurogenetics, University of British Columbia, Vancouver, Canada; 5 McKnight Brain Institute, University of Florida, Gainesville, USA

**Correction to: Mol Brain (2019) 12:92**

**https://doi.org/10.1186/s13041-019-0513-9**

Following publication of the original article [1], the authors reported errors in Fig. 4. Specifically, a wrong actin blot is presented in Fig. 4a. In this Correction, the corrected version of Fig. 4 is shown.

**Fig. 4:**
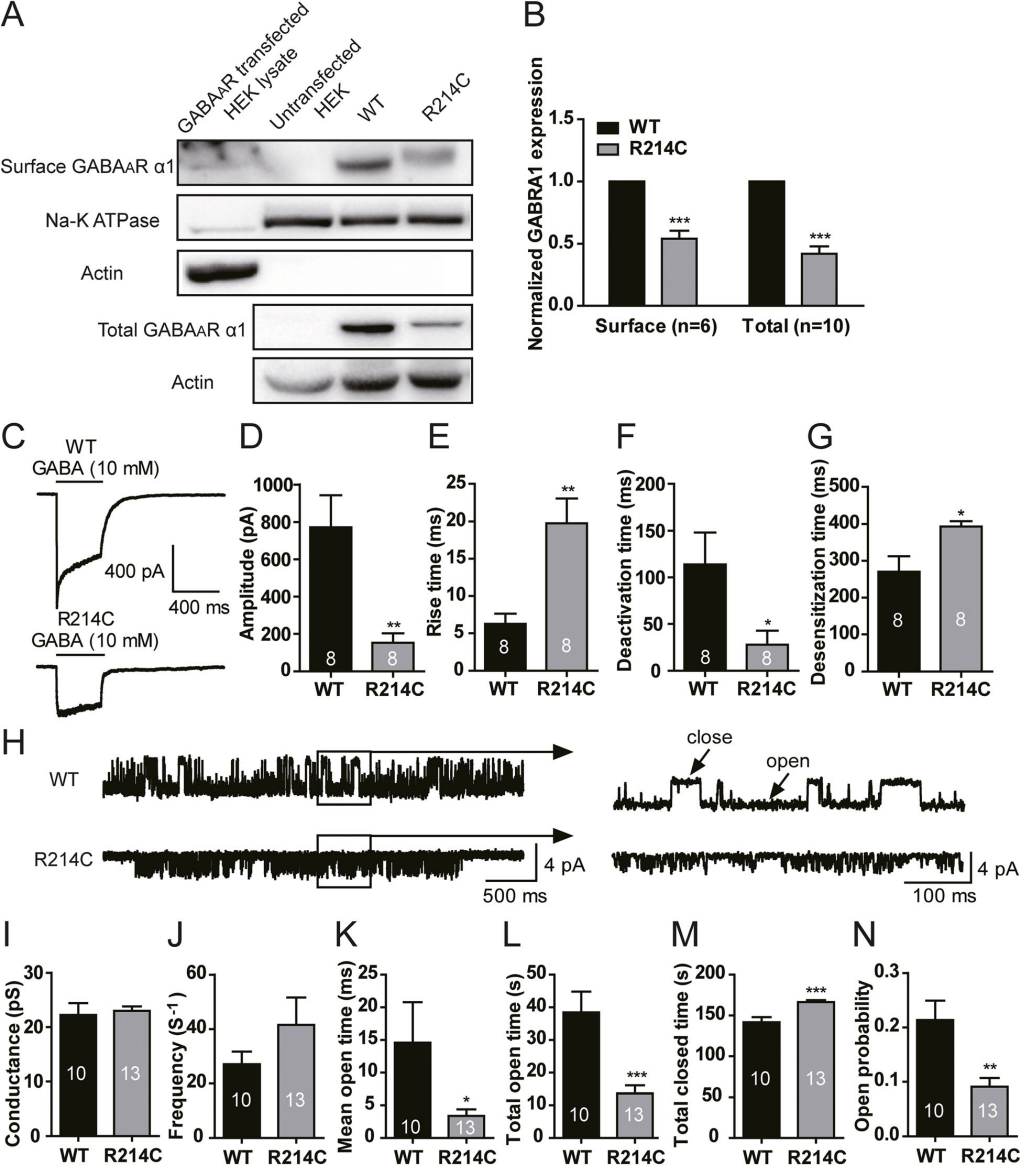
The R214C mutation resulted in reduced surface and total expression levels of the α1 subunit, and altered the kinetic and single channel properties of GABA_A_Rs. **a** Representative blots of biotinylation samples for surface receptor expression and cell lysates for total receptor expression from HEK293 cells expressing either WT or R214C GABA_A_Rs. **b** Quantification of surface α1 subunits normalized to Na^+^/K^+^ ATPase (*n* = 6), and total α1 subunits normalized to β-actin (*n* = 10). Statistical differences were determined using student’s *t*-test by comparing to expression levels of WT GABA_A_R expressing cells (***p < 0.001). **c** Representative traces of GABA currents recorded in excised macro-patch membrane under outside-out configuration from WT or R214C GABA_A_R expressing cells. Currents were evoked by rapidly perfusion of 10 mM GABA to the membrane patch for 400 ms. Quantification of averaged peak current amplitudes (**d**), 10–90% rise time (**e**), deactivation rate (**f**) and desensitization (**g**) in WT (*n* = 8) or R214C (*n* = 8) GABA_A_R expressing cells. **h** Representative single channel current traces recorded under cell-attached configuration with a pipette containing GABA (1 mM) at a holding potential of + 100 mV from cells expressing WT or R214C GABA_A_Rs. Quantified average of conductance (**i**), opening frequency (**j**), mean open time (**k**), total open time (**l**), total closed time (**m**), and open channel probability (**n**) of WT (*n* = 10) or R214C (*n* = 13) GABA_A_Rs. Statistical differences were determined using student’s *t*-test by comparing to WT GABA_A_R cells (*p < 0.05, **p < 0.01, ***p < 0.001)
