# Supplementary material for: Pathophysiology of and therapeutic options for a GABRA1 variant linked to epileptic encephalopathy
Source: Mol Brain. 2019 Nov 10;12:92. doi: 10.1186/s13041-019-0513-9 (PMC6842544; doi:10.1186/s13041-019-0513-9)
Supplement: Supplementary file 1 — Additional file 1: Figure S1. Verapamil induced maximum GABA-evoked chloride currents in R214C at 4 μM. [file 13041_2019_513_MOESM1_ESM.docx]

Additional Figures


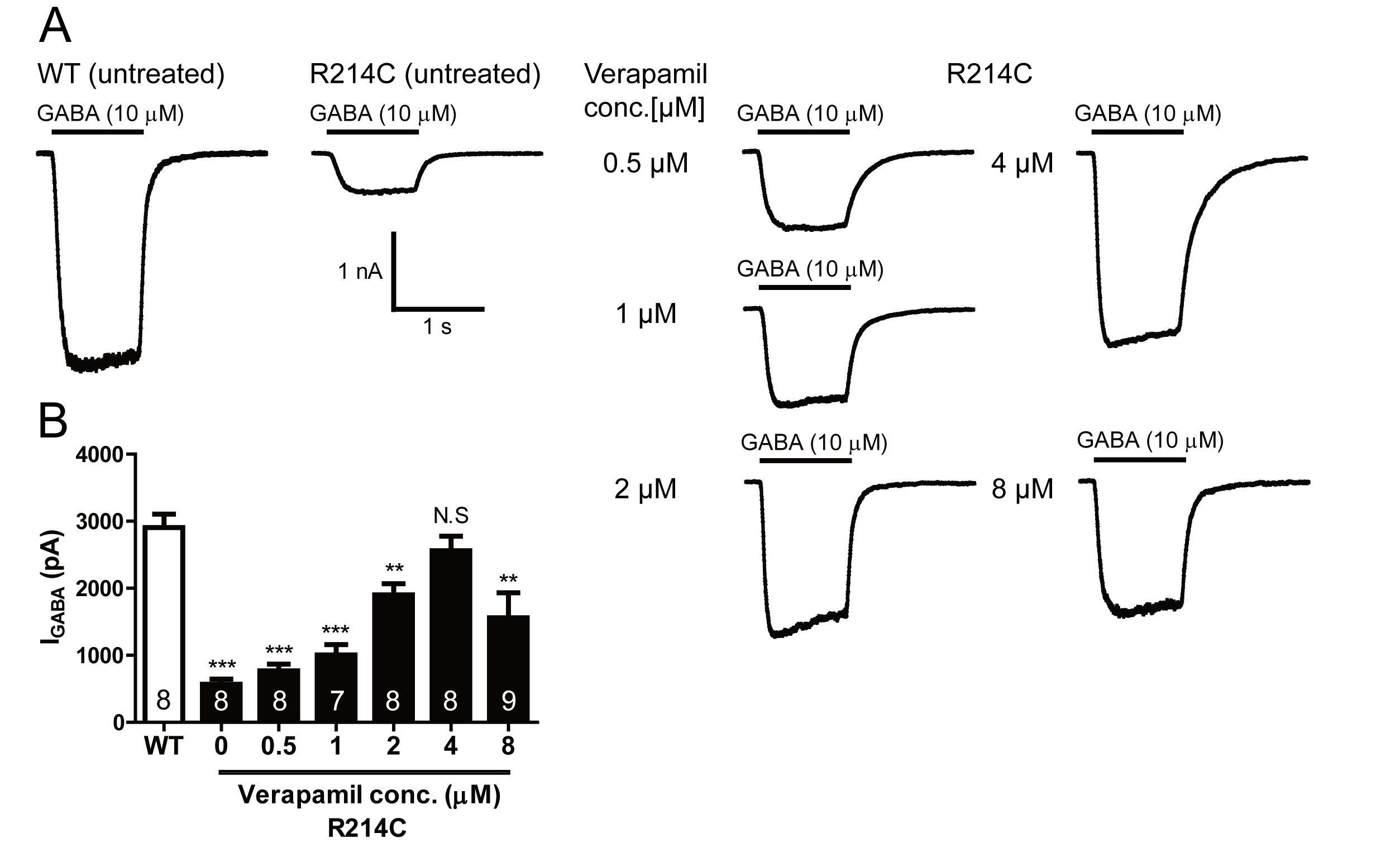


ADDITIONAL FILE 1: FIGURE S1: Verapamil induced maximum GABA-evoked chloride currents in R214C at 4µM. A. Representative traces of GABA-evoked currents from WT or R214C GABA_A_R expressing cells that were untreated (untreated) or incubated with verapamil at different concentrations (0.5µM-8µM, 24h). B. Quantification of averaged peak current amplitudes recorded from WT (n=8) or R214C (n=7-9) GABA_A_R expressing cells at indicated concentrations of verapamil. Statistical differences were determined using students *t*-test by comparing GABA-evoked currents to untreated WT (***p<0.01, ***p<0.001*). Data is represented as +SEM.
